# Perindopril treatment promote left ventricle remodeling in patients with heart failure screened positive for autoantibodies against angiotensin II type 1 receptor

**DOI:** 10.1186/1471-2261-13-94

**Published:** 2013-10-31

**Authors:** Qian Du, Jinling Wu, Hua Wang, Xin Wang, Lin Xu, Zhiyong Zhang, Jiamei Liu, Juan Zhang, Jin Chen, Hakon Hakonarson, Aihua Hu, Lin Zhang

**Affiliations:** 1 Heart Failure Center, Departments of Cardiology, Capital Medical University, Chao-Yang Hospital, Beijing, China; 2 Children's Hospital of Philadelphia Research Institute, University of Pennsylvania School of Medicine, Philadelphia, PA, USA

**Keywords:** Anti-AT_1_-AR, Renin-angiotensin-aldosterone system, Biomarker

## Abstract

**Background:**

Autoantibodies specific to the angiotensin II type I receptor (anti-AT_1_-AR) have been implicated in the pathology of congestive heart failure (CHF). Anti-AT_1_-AR may be associated with left ventricular function in CHF patients treated with perindopril.

**Methods:**

Synthetic angiotensin II type 1 receptor (AT_1_-R) peptides served as the target antigen. ELISA was used to screen the sera of 156 CHF patients, which were divided into positive and negative groups based on their anti-AT_1_-AR reactivity. Echocardiography and a 6-minute walk test were performed at baseline and after one year of perindopril therapy. The end-point events were compared over a 5-year follow-up.

**Results:**

Final analysis covered 138 patients, including 82 positive and 56 negative. The frequency and geometric mean titre of anti-AT_1_-AR were significantly lower in the positive group after one year of treatment (all *P* < 0.01, from 100% to 73.2% and from 1:125.3 ± 1.0 to 1:69.2 ± 1.1). Of these, 22 patients showed no antibodies. Both groups showed improvement in left ventricular end-diastole, end-systolic dimensions, ejection fraction, and a 6-minute walk test by perindopril in combination with standard treatment regime for one year (all *P* < 0.01). However, the 82 patients positive for anti-AT_1_-AR showed more pronounced improvement than the 56 negative patients (all *P* < 0.05). However, after 5 years of follow-up, the rate of all causes and cardiovascular mortality attributable to any cause and the re-hospitalisation rate showed no significant differences between the two groups (all *P* > 0.05).

**Conclusions:**

Perindopril treatment significantly decreased the frequency and geometric mean titre in patients positive for anti-AT_1_-AR, even to complete ablation. These patients showed greater improvement in left ventricular remodeling and heart function than negative that in patients after one year of perindopril treatment in combination with standard treatment, but no significant differences in endpoint events were observed in the following 5 years. Anti-AT_1_-AR might be a useful biomarker of over-activation of the renin-angiotensin-aldosterone system for clinical medication.

## Background

In recent years, there has been increased interest in autoimmune mechanisms involved in the pathogenesis of cardiovascular disease. Wallukat et al. demonstrated that the autoantibodies that activate angiotensin (Ang) II type 1 receptor (anti-AT_1_-AR) are similar to those observed for the natural agonist Ang II [1,2]. A previous report by the present team showed that anti-AT_1_-AR exists in the sera of congestive heart failure (CHF) patients with ischemic cardiomyopathy and hypertension and that it may play an important role in the pathogenesis and myocardial remodeling of heart failure. Recently, Jin et al. found that anti-AT_1_-AR causes changes in cardiac tissue and function in rats [3]. In vitro, mouse cells experience an agonist effect similar to Ang II in cardiomyocyte hypertrophy [4].

Perindopril, a long-acting angiotensin-converting-enzyme inhibitor, can block the conversion of Ang I to Ang II and decrease the effects of Ang II that are mediated by AT_1_ receptor binding. Several studies have demonstrated that long-term administration of angiotensin-converting-enzyme inhibitor and β- receptor blocker have additional therapeutic benefits [5,6]. However, the clinical value of anti-AT_1_-AR in heart failure patients and whether there are differential responses to perindopril between patients with and without anti-AT_1_-AR remains unknown.

In the present study it is hypothesised that CHF patients with anti-AT_1_-AR may experience more pronounced improvement in LV remodeling and heart function than patients without anti-AT_1_-AR in response to perindopril. Anti-AT_1_-AR may be useful as a marker for the pharmacological management in CHF patients.

## Methods

### Study population

This study was a prospective evaluation. The role of anti-AT_1_-AR was observed in CHF. From January 2005 to December 2010, 156 CHF patients with dilated cardiomyopathy (n = 38), ischemic cardiomyopathy (n = 58), and hypertensive heart disease (n = 60) were recruited for the study. All patients were evaluated with respect to symptoms, physical examination, laboratory tests, chest radiography, cardiograms, and echocardiography. Each patient performed a minimum of two times of 6-minute walk tests, and the first scores of the pre- and post-treatment walk tests served as baseline values [7].

Criteria for enrolment were as follows: (1) age 18–80 years old, (2) cardiac dysfunction caused by dilated cardiomyopathy, ischemic cardiomyopathy, or hypertensive heart disease, (3) stable New York Heart Association (NYHA) class II–IV heart function after treatment with β-receptor blocker, diuretics, and digoxin, (4) ability to complete the study visits, and (5) chronic cardiac insufficiency as defined by a LV ejection fraction (EF) ≤45%. Data from hospital and physician records were analysed for adverse events such as re-hospitalisation, death from cardiovascular causes, and death from other causes. Exclusion criteria were as follows: (1) bilateral renal artery stenosis, unilateral renal artery stenosis, loss of function of kidney, cardiogenic shock, angiotensin-converting-enzyme inhibitor hypersensitivity, (2) hepatic dysfunction, (3) haemoglobin, creatinine, glutamic pyruvic transaminase, and potassium above or below the normal limit, creatinine clearance ≤ 30 mL min^−1^ (calculated using the Cockcroft-Gault formula), (4) stroke within the past 3 months, (5) systolic blood pressure over 160 mmHg or diastolic blood pressure of more than 95 mmHg despite antihypertensive therapy, and (6) terminal disease with a predicted survival time <12 months (e.g. terminal cancer). The study is compliant with the Declaration of Helsinki and was approved by the Ethics Committee and the Prescription and Therapeutic Committee of Beijing Chao-yang Hospital-Affiliate of Capital of Medical University. All patients in this study were given informed consent prior to enrolment in the study.

### Serum sampling and peptide synthesis

Two millilitres of blood was drawn from the antecubital vein and separated by centrifugation (2000 rpm, Beckman CS-15R Centrifuge) for 10 min. Serum samples were stored at −20°C until needed for assay.

Peptides corresponding to the amino acid sequence (residues 165–191) of the second extracellular loop of the human AT_1_-AR (I-H-R-N-V-F-F-I-E-N-T-N-I-T-V-C-A-F-H-Y-E-S-Q-N-S-T-L) were synthesised commercially (Genemed Synthesis Inc. CA, U.S.) [8]. The purity of the peptides was determined by high-performance liquid chromatography (HPLC) (Vydac C-18 column) and direct sequence analysis with an automated amino acid analyser (Beckman Instruments Inc, Palo Alto, CA, U.S.).

### ELISA-based screening for anti-AT_1_-AR

Patients were defined as positive or negative subjects based on the presence or absence of anti- AT_1_-AR. An SA-ELISA protocol described previously was used to screen for the presence of the autoantibodies [8-11]. Briefly, 50 μL of the peptide (5 μg mL^−1^) in 100 mM Na_2_CO_3_ solution was coated on a microtitre plate overnight at 4°C. The wells were saturated overnight with phosphate-buffered saline (PBS) supplemented with foetal bovine serum (FBS, 10%, v/v). Serial dilutions of serum samples (1:20–1:160) were then added to the antigen-coated microtitre plates and stored overnight at 4°C. The blank group was given serum. An affinity-purified biotinylated rabbit antihuman lgG (H + L) antibody was allowed to react for 1 h. The wells were washed three times (PBS, 10% FBS and 0.1% Tween-20 v/v) and then incubated for 1 h with a streptavidin-peroxidase (2 μL mL^−1^) solution in PMT (PBS supplemented with 3% (wt/vol) of skimmed milk, 0.1% (v/v) of Tween-20 and 0.01% (wt/vol) of thimerosal (Sigma, St. Louis, MO, U.S.)). Incubation was followed by 3 rounds of washing with PBS, after which a peroxidase substrate (2.5 mm H_2_O_2_-2 mM 2, 2′-amino-di (2-ethyl-benzothiazoline sulphonic acid-6) ammonium salt (ABTS, Sigma, St. Louis, MO, U.S.) was added to the wells. The reaction was allowed to proceed for 30 min and then the sample’s photometric qualities were evaluated (optical density, at 405 nm) using a microplate reader (Labsystems Multiskan MK3, Helsinki, Finland).

### Administration of angiogenesis-converting-enzyme inhibitor

All patients received a standard pharmacological regimen for CHF (12.5–100 mg day^-1^ metoprolol, 10–20 mg Qd spironolactone, and 0.125–0.25 mg Qd digoxin) [12-14]. None of the patients included in this study had been treated with any angiotensin-converting-enzyme inhibitor before they enrolled in the study. Both groups received an initial daily dose of perindopril of 2 mg day^−1^ (2 mg Qd), then up-titred over a 2–4 week period to a target dose of 4 mg day^−1^ (4 mg Qd). The criteria for increasing the dose included systolic blood pressure of 90 mmHg or higher while the patient was standing, the absence of hypotension, and a serum creatinine concentration of either less than 2.0 mg per decilitre (177 μmol per litre) or no more than 50% higher than the baseline concentration. The target heart rate and blood pressure were 55 bpm and 90/60 mmHg, respectively. The 4 mg day^−1^ target dose was maintained until the termination of the study.

### Endpoint events and definitions

The primary endpoint events were all-cause and cardiovascular mortality for a protocol-specific cardiovascular case. Re-hospitalisation for any cardiovascular event was the secondary endpoint. All endpoint events were adjudicated by members of an independent endpoint committee who were unaware of study group assignments and used pre-specified criteria.

### Follow-up

Each patient was assigned to one designated study investigator from whom that patient received follow-up examinations at least once per month for 12 months after the initiation of the study, and then every 3 months for up to 5 years. Patients were encouraged to schedule interim appointments if needed. Data collected during patient examinations included heart rate, blood pressure, weight, presence of rales during the pulmonary exam, cardiac function, presence of peripheral edoema, and drug doses. Subjects were questioned and examined for the presence of any adverse drug reaction.

### Data analysis

All data except antibody titres are presented as mean values ± SEM. An anti-AT_1_-AR positive score was defined as a ratio P/N (sample OD − blank OD/negative control OD − blank OD) of ≥ 2.1. Negative scores were defined as a ratio P/N < 2.1. Antibody titres are reported as geometric means and differences between data sets were evaluated by unpaired Student’s t-tests.

All other data were evaluated by Fisher exact test measures. In all cases, a *P*-value of <0.05 was considered statistically significant, and survival analysis was performed over 5 years. Kaplan–Meier curves were analysed over time to determine probability of survival using the log rank test according to the presence or absence of these autoantibodies. Analyses were performed using the GraphPad 5.0 software package (San Diego, CA, U.S.).

## Results

### Clinical data and characteristics

The patients were assigned to the positive and negative groups based on anti-AT_1_-AR reactivity, 88 to the positive group and 68 to the negative group. Six patients in the positive group and twelve patients in the negative group were not enrolled in the study. Of these, three patients positive for anti-AT_1_-AR and four patients negative for anti-AT_1_-AR were not enrolled because their LVEF values improved to >45% after standard pharmacological intervention. One patient with (−) anti-AT_1_-AR experienced sudden death, two patients with (+) anti-AT_1_-AR and seven patients with (−) anti-AT_1_-AR withdrew, and one patient with (+) anti-AT_1_-AR ceased contact with the study personnel. Finally, there were 138 patients in this study, 82 patients with (+) anti-AT_1_-AR and 56 patients with (−) anti-AT_1_-AR. The mean disease history (years) of (+) anti-AT_1_-AR patients were shorter than those of (−) anti-AT_1_-AR patients (*P* < 0.01, 6.1 ± 0.3 vs. 8.7 ± 0.4 years). Other baseline clinical characteristics remained consistent across all patients in the study (Table 1).

**Table 1:** Clinical characteristics of patients (means ± SEM)

**Index**	**(+) anti-AT**_**1**_**-AR**	**(−) anti-AT**_**1**_**-AR**	***P*-value**
	**(n = 82)**	**(n = 56)**	
**Demographic**			
Age (years)	66.6 ± 1.2	65.1 ± 1.6	ns
Sex (male)	42 (51.2%)	30 (53.6%)	ns
BMI (kg/m^2^)	24.3 ± 0.9	24.3 ± 0.6	ns
Disease history (years)	6.1 ± 0.3	8.7 ± 0.4	<0.01
**Aetiology**			
CAD cases (%)	24 (29.3%)	26 (46.4%)	ns
DCM cases (%)	26 (31.7%)	8 (14.3%)	ns
HHD cases (%)	32 (39.0%)	22 (39.3%)	ns
**Physiological characteristics**			
Heart rate (bpm)	94.2 ± 1.2	93.8 ± 1.2	ns
SBP (mmHg)	129.9 ± 1.8	125.0 ± 1.8	ns
DBP (mmHg)	78.2 ± 0.9	75.5 ± 1.1	ns
LVEDD (mm)	69.5 ± 0.6	69.9 ± 0.9	ns
LVESD (mm)	56.2 ± 0.6	55.7 ± 0.8	ns
LVEF (%)	34.1 ± 0.6	33.8 ± 0.9	ns
6 min walk test (m)	303.8 ± 3.7	301.8 ± 4.3	ns

CAD: coronary artery disease; DCM: dilated cardiomyopathy; HHD: hypertensive heart disease; SBP: systolic blood pressure; DBP: diastolic blood pressure; LVEDD: left ventricular end-diastolic dimension; LVESD: left ventricular end-systolic dimension; LVEF: left ventricular ejection fraction.

### Frequency of anti-AT_1_-AR in the positive group

The frequency of anti-AT_1_-AR decreased significantly in the positive group (*P* < 0.01, from 100% to 73.2%). Twenty two patients demonstrated complete ablation of anti-AT_1_-AR titres one year after the initiation of perindopril treatment. The geometric mean titre of the remaining 60 patients in the (+) anti-AT_1_-AR group was significantly lower than baseline values. The geometric mean titres were reduced as follows: Twenty one patients went from 1:160 to 1:80; ten patients went from 1:160 to 1:40; four patients went from 1:160 to 1:20; and two patients went from 1:80 to 1:40; one patient went from 1:80 to 1:20, and two patients went from 1:40 to 1:20. However, the remaining 20 patients experienced no change (Table 2). The current results provide clear clinical evidence that the anti-AT_1_-AR produced by CHF patients is suppressed by treatment with perindopril.

**Table 2:** **Perindopril and frequency and titre of anti-AT**_**1**_**-AR**

	**Pre-treatment**	**Post-treatment**	***P*-value**
	**n = 82**	**n = 58**	
AT_1_ autoantibody frequency (%)	100%	70.7%	<0.01
Mean titre	125.3 ± 1.0	69.2 ± 1.1	<0.01
Titre 1 : 160	55	13	<0.01
Titre 1 : 80	25	27	ns
Titre 1 : 40	2	12	ns
Titre 1 : 20	0	6	ns

Mean titre values are presented as geometric means ± SEM.

### Echocardiography and the 6-minute walk test

After one year of perindopril treatment in combination with standard treatment for CHF (β-receptor blockers, digoxin, and diuretics), LVEDD and LVESD values decreased from 69.5 ± 0.6 to 58.0 ± 0.8 mm and 56.2 ± 0.6 to 42.0 ± 0.7 mm, respectively, in patients with (+) anti-AT_1_-AR and from 69.5 ± 0.6 to 58.0 ± 0.8 mm and 56.2 ± 0.6 to 42.0 ± 0.7 mm, respectively, in patients with (−) anti-AT_1_-AR. Similarly, LVEF increased from 34.1 ± 0.6 to 49.6 ± 1.2% in patients with (+) anti-AT_1_-AR and from 33.8 ± 0.9 to 44.3 ± 1.1% in patients with (−) anti-AT_1_-AR (all *P* < 0.01).

It is worth noting that patients with (+) anti-AT_1_-AR showed greater improvements with respect to in LV remodeling and heart function than patients with (−) anti-AT_1_-AR (all *P* < 0.05), as shown in Table 3.

**Table 3:** Perindopril and echocardiographic data

	**(+) anti- AT**_**1**_**-AR (n = 82)**		**(−) anti- AT**_**1**_**-AR (n = 56)**	
	**Baseline**	**One-year**	**Baseline**	**One-year**
LVEDD (mm)	69.5 ± 0.6	58.0 ± 0.8*†	69.9 ± 0.9	61.0 ± 0.6*
LVEDD (mm)	56.2 ± 0.6	42.0 ± 0.7*†	55.7 ± 0.8	47.1 ± 1.0*
LVEF (%)	34.1 ± 0.6	49.6 ± 1.2*†	33.8 ± 0.9	44.3 ± 1.1*

LVEDD, left ventricular end-diastolic dimension; LVESD, left ventricular end-systolic dimension; LVEF, left ventricular ejection fraction.

Both groups of patients showed significant improvement in response to perindopril therapy. Of note, the presence of (+) anti-AT_1_-AR group experienced more pronounced benefits than the (−) anti-AT_1_-AR group. Data were collected and analysed as described in the text. (*P < 0.01 vs. baseline and †*P* < 0.05 vs. (−) anti-AT_1_-AR).

The 6-minute walk distance scores of study participants at the beginning and experimental endpoint of the investigation were illustrated. Perindopril in combination with standard treatment was associated with significant increases in the distance covered in a timed 6-minute walk in both groups. The patients with (+) anti-AT_1_-AR distance walked increased from 303.8 ± 3.7 to 488.6 ± 11.2 m (*P* < 0.01) and the patients with (−) anti-AT_1_-AR was from 301.7 ± 4.3 to 452.9 ± 9.2 m (*P* < 0.01), but the former group showed more pronounced improvement (*P* <0.05, 488.6 ± 11.2 vs. 452.9 ± 9.2 m). Clinical lab data including haemoglobin, creatinine, glutamic pyruvic transaminase, and potassium levels remained stable throughout the first year.

### Tolerability and maximal titration of perindopril

**Figure 1:**
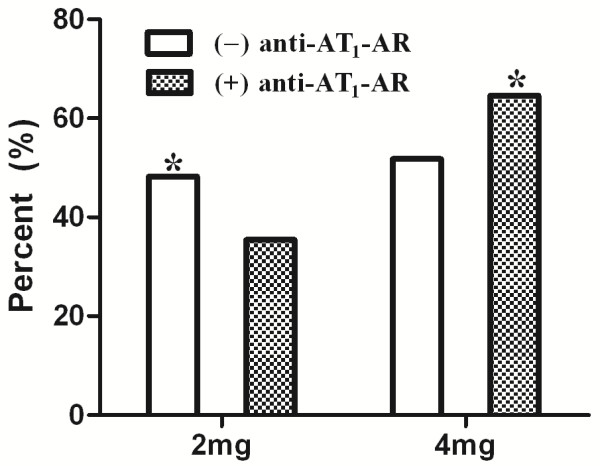
**Perindopril dosage.** During the one-year treatment period, the mean perindopril dose in the (+) anti-AT_1_-AR and (−) anti-AT_1_-AR group was 3.3 ± 0.1 mg Qd /day and 2.9 ± 0.1 mg Qd/day (*P* <0.05), respectively. Perindopril (4 mg) was administered to patients with (+) anti-AT_1_-AR at significantly higher doses than patients with (−) anti-AT_1_-AR (*P* < 0.05, 64.6% vs. 44.6%). These results suggest that the patients with (+) anti-AT_1_-AR are more tolerant to perindopril than those that do not express these autoantibodies.

During the one-year treatment period, the mean perindopril doses for individuals the (+) anti-AT_1_-AR and (−) anti-AT_1_-AR were 3.3 ± 0.1 mg per/day and 2.9 ± 0.1 mg per/day (*P* < 0.05), respectively. Perindopril (4 mg) was administered to at least 64.6% (53/82) of (+) anti-AT_1_-AR patients, significantly more than the number of (−) anti-AT_1_-AR patients (44.6%, 25/56, *P* < 0.05). These results suggested that the patients with (+) anti-AT_1_-AR demonstrated a greater tolerance to perindopril than those who did not express the autoantibodies (Figure 1).

### Primary endpoint events

**Figure 2:**
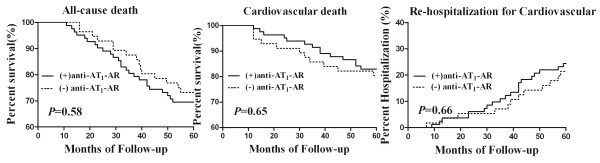
**Endpoint events in both groups over 5 years.** There was no significant difference in total mortality, cardiovascular mortality, or re-hospitalisation for a protocol-specific cardiovascular cases between patients with (+) anti-AT_1_-AR and (−) anti-AT_1_-AR (all *P* > 0.05).

Mortality from any cause, cardiovascular mortality, and the re-hospitalisation rate in the (+) anti-AT_1_-AR group were 17.1% (14/82), 30.5% (25/82), and 14.4% (20/82) respectively. These values were not significant different from the 19.6% (11/56), 26.8% (15/56), and 21.4% (12/56) recorded for the (−) anti-AT_1_-AR group (*P* = 0.58, *P* = 0.65, and *P* = 0.66 respectively), as shown in Figure 2.

### Adverse effects

One year of treatment with perindopril showed no significant effect on liver or kidney function. Three subjects became lethargic during the course of incremental administration of perindopril. However, these symptoms subsided as treatment continued. At least 3.6% (5/138) of the patients developed a cough due to perindopril and were then transitioned to angiotensin receptor antagonist therapy with losartan.

## Discussion

### Main findings

In this study, three novel observations were made. First, the frequency and geometric mean titre of anti-AT_1_-AR deceased significantly in positive group. Patients with (+) anti-AT_1_-AR showed greater improvements in LV remodeling and heart function than patients without anti-AT_1_-AR. Second, patients with (+) anti-AT_1_-AR were able to tolerate higher doses of perindopril than patients with (−) anti-AT_1_-AR during the first year of follow-up. Third, no statistically significant difference was detected between the two groups in terms of mortality from all cause, cardiovascular mortality, or re-hospitalisation after five years of follow-up. These results suggested that perindopril may partially and selectively inhibit or block over-activation of AT_1_-receptor by anti-AT_1_-AR. Furthermore, the long-term efficacy of perindopril in patients with (+) anti-AT_1_-AR was found to be equivalent to those patients with (−) anti-AT_1_-AR, and the reasons for this may involve patients with (+) anti-AT_1_-AR becoming negative.

### Role of anti-AT_1_-AR in heart failure

In recent years, evidence showing that an autoimmune mechanism might contribute to the pathogenesis of heart failure has accumulated. It has been reported that anti-AT_1_-AR, anti-β_1_-AR, and anti-M_2_-R are present in sera of patients with a variety of cardiovascular diseases [9,10,15]. Recently Jin et al. found that, in rats, anti-AT_1_-AR could cause changes in cardiac tissue and function and that it exhibited an agonist effect in vitro similar to Ang II in cardiomyocyte hypertrophy [3]. Anti-AT_1_-AR is involved in the pathogenesis of cardiovascular diseases. In this way, the elimination of these antibodies might have therapeutic benefit for CHF patients.

### Anti-AT_1_-AR and activation of RAAS

CHF is attributed to the long-term effects of cardiac ventricular reconstitution and excessive activation by neuroendocrine hormones, especially in the renin-angiotensin-aldosterone system (RAAS) [16]. Some studies have shown that cardiac AT_1_-AR is the primary receptor behind the regulation of heart function and that it mediates most of the known chronotropic and inotropic effects of Ang II in the heart, presynaptic facilitation of noradrenaline release from cardiac sympathetic nerve terminals, coronary vessel vasoconstriction, stimulation of aldosterone release, myocyte hypertrophy, non-myocyte proliferation, and interstitial fibrosis [17,18]. Anti-AT_1_-AR has also been showed to perform functions similar to those of Ang II [19,20]. The production of anti-AT_1_-AR may modulate the pathophysiological process of CHF by activating the RAAS and promoting ventricular remodeling in an angiotensin-II-independent manner. The binding of anti-AT_1_-AR to AT_1_-AR can activate cell signalling pathways involved in cell damage, proliferation, and other pathophysiological processes [21]. One recent study demonstrated anti-AT_1_-AR can induce apoptosis of neonatal rat cardiomyocytes in a dose- and time-dependent manner. Anti-AT_1_-AR has been shown to increase TNF secretion and caspase-3 activity. AT_1_ receptor blockage completely abrogated anti-AT_1_-AR-induced TNF-α secretion, caspase-3 activation, and cardiomyocyte apoptosis [22]. The existence of an autoimmune-activating receptor, such as anti-AT_1_-AR, could contribute to Ang II sensitivity [23]. These parameters may also be useful in the diagnosis and assessment of the pathogenesis of heart failure and in the stratification and identification of patients who would be at higher risk of heart failure or more responsive to therapeutic agents [24,25].

### Perindopril and the effects of anti-AT_1_-AR

Perindopril belongs to the angiotensin-converting enzyme (ACE) inhibitor class of medications. It is a potent, competitive inhibitor of ACE, the enzyme responsible for the conversion of angiotensin I (AT_I_) to angiotensin II (AT_II_), which regulates blood pressure and heart function. AT_II_ is a key component of the renin-angiotensin-aldosterone system (RAAS). It is here speculated that perindopril may be directly or indirectly related to the inhibition of over-activity of RAAS. Perindopril may decrease Ang II and increase Ang-(1–7) levels and so may attenuate immune responses to reduce or abolish the anti-AT_1_-AR effects. Angiotensin Ang-(1–7) is a biologically active peptide of the renin-angiotensin system whose actions are often opposite to those attributed to Ang II. This peptide may play a part in the beneficial effects of angiotensin-converting enzyme inhibitors in cardiovascular disease [26]. Tallant et al. reported that Ang-(1–7) inhibits the growth of cardiomyocytes and is associated with reduced activity of MAP kinase. These responses are opposite those of Ang II-AT_1_ activation. Cardiofibroblasts and Ang-(1–7) may underlie the anti-inflammatory actions of the peptide that reverse cardiac fibrosis in the DOCA-salt rat model [27]. Ang-(1–7) induce vasodilatation, exert antiproliferative effects in vascular smooth muscle cells, and increase the sensitivity of the baroreflex.

Extensive studies have shown that AT_2_-receptor is re-expressed by cardiac fibroblasts during heart failure. Direct AT_2_ receptor stimulation was found to induce anti-inflammatory and anti-oxidant effects and was involved in anti-remodeling mechanisms that improved cardiac function [28,29]. It is here speculated that anti-AT_1_-AR and Ang II may stimulate re-expression of the AT_2_ receptor in some situations to some extent and that this might have therapeutic effects. Moreover, in primary aldosteronism, after challenge with ACE inhibitors, the plasma aldosterone concentration fell more in anti-AT_1_-AR positive individuals than in negative individuals, suggesting that this autoantibody plats an agonistic role [30]. ACE inhibitors may reduce plasma aldosterone through anti-AT_1_-AR agonism. Therapies for heart failure and activation of the immune system may improve as drug standardisation becomes more sophisticated, and antibody titre should decrease.

### Predictive value of anti-AT_1_-AR

Continual activation of autoantibodies may lead to a poor prognosis. Studies have shown that stimulating autoantibodies against the cardiac β1-adrenergic receptor can predict increased mortality among individuals with idiopathic cardiomyopathy [31]. It is here suggested that perindopril may weaken or even eliminate the adverse effects of anti-AT_1_-AR during the initial stage of CHF. This might be mediated by activating an immune mechanism that can weaken or offset CHF and ventricular remodeling and so improve cardiac function. In this way, long-term administration of perindopril in conjunction with digoxin, diuretics, and beta-blockers may prevent over-stimulation of AT_1_-AR by anti-AT_1_-AR and reduce the progression of cardiac damage. In addition, perindopril, which is an angiotensin-converting-enzyme inhibitor, can decrease plasma aldosterone levels and improve the prognosis of patients with severe CHF by decreasing plasma rennin-angiotensin levels [32]. In this way, anti-AT_1_-AR may be used to predict treatment response to select angiotensin-converting-enzyme inhibitors in patients with CHF. The specific pathological mechanism of anti-AT_1_-AR requires further exploration.

## Conclusions

The results of this study suggest that the frequency and geometric mean titre in patients with (+) anti-AT_1_-AR decreases significantly, even complete ablation. Patients positive for anti-AT_1_-AR experience more pronounced improvements in left ventricular function and remodeling than patients without anti-AT_1_-AR when given perindopril in combination with standard treatment over a shorter period of time. It should be noted that long-term clinical efficacy in patients with (+) anti-AT_1_-AR was equivalent to that observed in patients without anti-AT_1_-AR. It is here speculated that anti-AT_1_-AR might be a useful biomarker of over-activation of the renin-angiotensin-aldosterone system. This is the first study to link anti-AT_1_-AR to treatment strategies for congestive heart failure.

## Competing interests

The authors declare that they have no competing interests.

## Authors’ contributions

LZ contributed to study design and drafting part of manuscript. All authors contributed to the acquisition of data and analysis of the results. QD, WJL, WH, and LZ were involved in drafting the manuscript. All authors revised the manuscript critically and approved the final version.

## Pre-publication history

The pre-publication history for this paper can be accessed here:

http://www.biomedcentral.com/1471-2261/13/94/prepub
